# A Case Report of Hemorrhagic Cavernoma Masquerading as a Thrombosed Aneurysm

**DOI:** 10.7759/cureus.50548

**Published:** 2023-12-14

**Authors:** Nadeen Boubshait, Hassan Alhassar, Noufa Alsubaie, Abdulla Jan, Farah Asad

**Affiliations:** 1 General Practice, King Faisal University, Hofuf, SAU; 2 General Practice, Imam Abdulrahman Bin Faisal University, Dammam, SAU; 3 Family Medicine, University of Jeddah, Jeddah, SAU; 4 Public Health, Primary Health Care Center, Hofuf, SAU

**Keywords:** cerebral cavernoma, cerebral aneurysm, digital subtraction angiography, headache, generalized seizure, magnetic resonance imaging

## Abstract

Cavernous malformations are vascular lesions characterized by dilated blood vessels with minimal intervening brain parenchyma. Although often asymptomatic, they can present with seizures, headaches, or neurological deficits. Accurate diagnosis relies on magnetic resonance imaging, with characteristic features such as a "popcorn" appearance. We present a case of a 45-year-old male with chronic headaches and seizures who underwent an extensive work-up. Initial magnetic resonance imaging suggested a thrombosed aneurysm, with subsequent cerebral angiography being unremarkable, supporting the final diagnosis of a cavernous malformation. Conservative management, initiated for asymptomatic lesions, led to effective seizure control and improved quality of life. This case underscores diagnostic complexities in neuroradiology, emphasizing the need for careful consideration of differentials when faced with unexpected imaging results. Clinicians must remain vigilant for alternative explanations, recognizing the dynamic nature of optimal strategies in neurovascular medicine.

## Introduction

Cavernous malformations, also known as cavernomas or cavernous angiomas, are vascular lesions characterized by clusters of dilated, thin-walled blood vessels with minimal intervening brain parenchyma. While often asymptomatic, these lesions may manifest with seizures, headaches, or neurological deficits when situated in critical brain regions. Magnetic resonance imaging serves as the cornerstone for diagnosing cavernomas, revealing a characteristic "popcorn" appearance with a rim of signal loss attributed to hemosiderin [1]. However, the potential overlap in radiological features with other pathologies necessitates careful evaluation.

Thrombosed aneurysms pose a diagnostic challenge due to their diverse imaging characteristics. Accurate differentiation between thrombosed aneurysms and other lesions is crucial for directing patient management and treatment strategies [2]. This case report describes a patient initially suspected to have a thrombosed aneurysm based on magnetic resonance imaging findings. However, further investigation revealed a hemorrhagic cavernoma after a normal digital subtraction angiography scan.

## Case presentation

A 45-year-old male sought medical attention at the neurology clinic with a six-month history of chronic headaches characterized by throbbing pain in the left temporal region, occasionally accompanied by nausea. Despite an initial diagnosis of chronic migraines, the patient returned two months later after experiencing a witnessed generalized tonic-clonic seizure.

Neurological examination findings revealed mild left-sided facial weakness (House-Brackmann Grade II) and a subtle reduction in sensation along the left side of the face. Additionally, there was evidence of a subtle left-sided pronator drift and mild dysmetria on finger-to-nose testing. Following this development, an extensive work-up was initiated.

**Figure 1:**
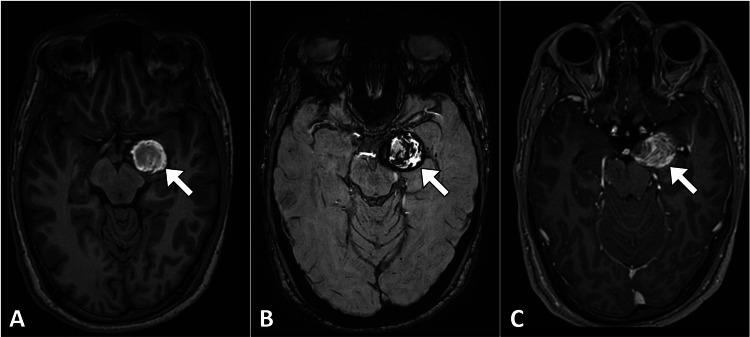
Axial MR images of the brain illustrating a lesion in the medial aspect of the left temporal lobe. The lesion displays high signal intensity on the T1-weighted image (A), a peripheral blooming artifact on the gradient-echo image (B), and faint contrast enhancement (C). MR: Magnetic resonance

Laboratory investigations, including complete blood count, electrolytes, liver function tests, and coagulation profile, were unremarkable. An electroencephalogram revealed abnormal focal epileptiform discharges in the left temporal region. An urgent magnetic resonance imaging of the brain was ordered, revealing a lesion close to the M1 segment of the left middle cerebral artery. The initial interpretation of the magnetic resonance imaging findings raised concerns of a thrombosed aneurysm, as the lesion demonstrated high signal intensity on both T1-weighted and T2-weighted images. Notably, a gradient echo image revealed a blooming artifact, suggestive of hemorrhage within the lesion. Furthermore, the presence of a mass effect on the left cerebral peduncle was observed, emphasizing the potential impact of the lesion on adjacent brain structures (Figure 1).

**Figure 2:**
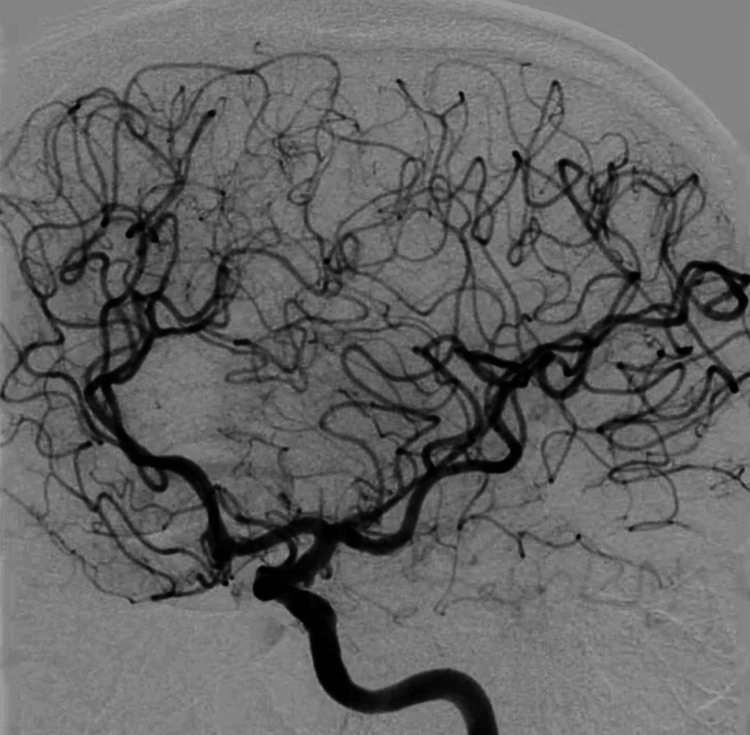
Digital subtraction angiography of the left internal carotid artery, revealing the absence of any detectable aneurysms.

To further characterize the vascular nature of the lesion and confirm or exclude the possibility of a thrombosed aneurysm, digital subtraction angiography cerebral angiography was performed. Surprisingly, the digital subtraction angiography results were unremarkable, revealing no evidence of vascular abnormality (Figure 2). This unexpected outcome prompted a revised interpretation of the magnetic resonance imaging findings, attributing them to a hemorrhagic cavernoma rather than a thrombosed aneurysm.

Given the absence of significant neurological deficits, a conservative management approach was chosen. The patient was initiated on antiepileptic medication for seizure control, and close monitoring was instituted. The hospital course remained uneventful, with no further seizures reported. Follow-up visits at regular intervals demonstrated effective control of headaches and seizures with the prescribed medication, leading to a significant improvement in the patient's quality of life.

## Discussion

The presented case sheds light on the diagnostic challenges encountered in differentiating cavernous malformations from other mass lesions, such as thrombosed aneurysms. Cavernomas represent vascular anomalies characterized by clusters of dilated, thin-walled blood vessels with minimal intervening brain parenchyma. While often asymptomatic, these lesions may manifest with seizures, headaches, or neurological deficits when located in critical brain regions. The diagnostic cornerstone for cavernomas is magnetic resonance imaging, particularly utilizing gradient-echo sequences and susceptibility-weighted imaging sequences. These sequences reveal the characteristic "popcorn" appearance with a rim of signal loss due to hemosiderin deposition, providing essential insights for accurate characterization [1]. In our case, the initial suspicion of a thrombosed aneurysm based on magnetic resonance imaging findings and subsequent confirmation of a cavernous malformation through digital subtraction angiography accentuates the complexities involved in neuroimaging interpretation.

The normalcy of the digital subtraction angiography results, in this case, may be attributed to the slow blood flow and low-pressure nature of the dilated, thin-walled vessels comprising the cavernous malformation. Given these hemodynamic characteristics, it is conceivable that the vessels filled too slowly to exhibit adequate contrast in the angiogram. The discrepancy between the highly suggestive magnetic resonance imaging findings and the apparently normal DSA results highlights the need to recognize the limitations of imaging modalities. The timing of contrast injection during DSA, coupled with the slow-filling dynamics of cavernous malformations, could contribute to the lack of conspicuous contrast enhancement in the angiographic study.

The discussion extends to the differential diagnosis of cavernous venous malformations, which includes consideration of other cerebral vascular malformations, such as arteriovenous malformations, venous angiomas, and capillary telangiectasias. Dural arteriovenous fistulas, aneurysms, vein of Galen malformations, and hemorrhagic or calcified neoplasms are also part of the comprehensive differential considerations. Additionally, inflammatory or infectious masses, granulomas, subacute hematomas, cerebral amyloid angiopathy, hemorrhagic cerebral metastases, chronic hypertensive encephalopathy, cerebral vasculitis, and tuberculoma may present with features that necessitate careful differentiation from cavernous venous malformations [1,3].

Cerebral cavernomas have a spectrum of management options, primarily encompassing conservative care, microsurgical excision, and stereotactic radiosurgery. The decision-making process is intricate, taking into consideration factors such as the natural history of cavernous malformations, clinical presentation, lesion location, frequency of hemorrhagic episodes, and existing medical conditions [3,4].

Conservative management, involving periodic imaging and monitoring, is often chosen for asymptomatic cavernous malformations or those in low-risk locations. This approach aims to minimize potential intervention-related risks, particularly when lesions are incidentally discovered and do not cause significant neurological impairments. Microsurgical excision becomes a consideration, especially for cases linked to medically resistant epilepsy where the epileptogenic focus can be attributed to the cavernoma. The decision to opt for surgery is carefully weighed against potential benefits, considering the patient's overall clinical status and the impact of the cavernous malformation on neurological function. Stereotactic radiosurgery, while less common, is reserved for surgically challenging cases or lesions in critical brain regions, delivering focused radiation to induce changes in the cavernoma vasculature and reduce the risk of hemorrhage over time. The choice of management is individualized, reflecting the unique characteristics of each case and the patient's overall health, while ongoing research contributes to the dynamic evolution of optimal strategies in neurovascular medicine [5].

## Conclusions

In conclusion, this case underscores the diagnostic challenges in neuroradiology, exemplified by the initial misinterpretation of a thrombosed aneurysm, which was corrected upon further evaluation, highlighting the complexity of neuroimaging interpretation. The case also emphasizes the need to consider a broad range of differentials when faced with unexpected imaging results and stresses the importance of clinicians remaining vigilant for alternative diagnoses. The patient's favorable outcome with conservative management further supports the importance of tailored treatment strategies based on accurate diagnoses.

## References

[1] Wang KY, Idowu OR, Lin DD (2017). Radiology and imaging for cavernous malformations. Handb Clin Neurol.

[2] Kim YJ, Jeun SS, Park JH (2015). Thrombosed large middle cerebral artery aneurysm mimicking an intra-axial brain tumor: case report and review of literature. Brain Tumor Res Treat.

[3] Snellings DA, Hong CC, Ren AA (2021). Cerebral cavernous malformation: from mechanism to therapy. Circ Res.

[4] Akers A, Al-Shahi Salman R, A Awad I (2017). Synopsis of guidelines for the clinical management of cerebral cavernous malformations: consensus recommendations based on systematic literature review by the Angioma Alliance Scientific Advisory Board clinical experts panel. Neurosurgery.

[5] Hill CS, Borg A, Horsfall HL, Al-Mohammad A, Grover P, Kitchen N (2023). Cerebral cavernous malformation: management, outcomes, and surveillance strategies - a single centre retrospective cohort study. Clin Neurol Neurosurg.

